# Capacity Models and Transmission Risk Mitigation: An Engineering Framework to Predict the Effect of Air Disinfection by Germicidal Ultraviolet Radiation

**DOI:** 10.6028/jres.126.057

**Published:** 2022-03-21

**Authors:** Sam Rhea Sarcia

**Affiliations:** 1 Framework Product Development, Lakeside, CA 92040, USA

**Keywords:** air disinfection, efficiency, modelling, risk management, ultraviolet germicidal irradiation, UVGI, Wells-Riley

## Abstract

A first-principles–based model for predicting the effect of germicidal radiation interventions for air disinfection is presented. Calculation of the “capacity” of an intervention expressed in volumetric flow rate allows for a direct comparison against fresh-air dilution ventilation and filtration systems, which are quantified in terms of the clean air provided. A closed-form expression to predict the combined quantitative impact of spatial gradients and mixing currents on the efficiency with which an intervention is applied is introduced. If validated, this would allow for systems to be selected and sized based on simple metrics across a broad range of settings and applications. The expressions developed are compared against available experimental data sets, and future validation efforts are proposed. Additionally, a method to identify an optimal operating capacity for a given setting by comparing costs associated with disease transmission against the cost of capacity is derived using the Wells-Riley equation and presented as an appendix.

## 1 Introduction

The coronavirus disease 2019 (COVID-19) pandemic has provided further evidence that aerosol transmission of respiratory-disease-causing pathogens, such as severe acute respiratory syndrome coronavirus 2 (SARS-CoV-2) and influenza, is a greater threat than previously acknowledged [1]. Unlike fomite and droplet transmission, aerosol transmission can occur across long distances and over long time periods, making it less susceptible to controls such as social distancing and hand or surface sanitization [2]. The Wells-Riley equation, shown in Eq. (1), is a widely accepted epidemiological model used to predict transmission risk of airborne pathogens. It shows that the risk of disease transmission in a shared space is dependent on a series of variables, including the number of infected individuals present, *I*, the infectiousness and strength of the source(s), *q*, the inhalation rate, *p*, the exposure time, *t*, and the pathogen- removal capacity, *Q*, in units of clean-air flow rate [3].

probability of transmission = 1-$e\left(\frac{-Iqpt}{Q}\right)$(1)

Airborne pathogens may be removed from a space using fresh-air ventilation and filtration and may also be inactivated in place through exposure to germicidal irradiation in the ultraviolet-C (UV-C) spectrum (between 200 nm and 280 nm), which is equivalent in effect to physical removal and may be quantified similarly. An optimized risk-mitigation strategy for a given indoor setting will involve balancing the cost of disease transmission against the cost of pathogen-removal capacity while minimizing the specific cost of said capacity. In Appendix B (Sec. 9), the Wells-Riley equation is used to facilitate such an optimization process. UV-C inactivation has been shown to be the most cost-effective solution available today in this regard, but it is not well characterized and remains underutilized as a risk-mitigation mechanism [4]. One reason for this situation is the lack of sizing tools that are adaptable to a variety of pathogens and settings, and simple enough for application in a typical building-technology workflow, which is due to the complexities associated with the ways in which radiation spreads and air mixes in a room. A framework is presented here that is based on well-established first-principles relationships and attempts to distill relatively complex engineering equations into a form that a variety of stakeholders can use when deploying UV-C systems for the purpose of removing airborne pathogens.

## 2 Glossary of Variables

*I*= *number of infected individuals* *q*= *quanta generation rate* (*1/s*)
*p*= *respiration rate* (*m*^3^*/s*) *t*= *time* (*s*)
*Q*= *pathogen-rempoval rate* (*m*^3^*/s*) *N*= *pathogen concentration* (*%*)
*V*= *room volume*(*m*^3^) *E*= *irradiance* (*W/m*^2^)
*Z*= *susceptibility* (*m*^2^*/J*) *P* = *optical power* (*W*)
*L*= *beam length* (*m*) *A* = *wave front area* (*m*^2^)
*OC* = *optical capacity GC* = *germicidal capacity* (*m*^3^*/s*)
*DC* = *disinfection capacity* (*m*^3^*/s*) *DE* = *delivers efficiency* (*%*)
*SPE* = *spatial efficiency n* = *volume fraction denominator*
*C*_*mix*_= *mixing coefficient* (*1/h*) *V*_*mix*_ = *room airspeed* (*m/s*)
*L*_*room*_= *room linear dimension* *V*_*c*_ = *air cleaner volume* (*m*^3^)
*X *= *mixing coefficient scaler*

## 3 Dilution Ventilation and Transmission Risk

**Fig. 1:**
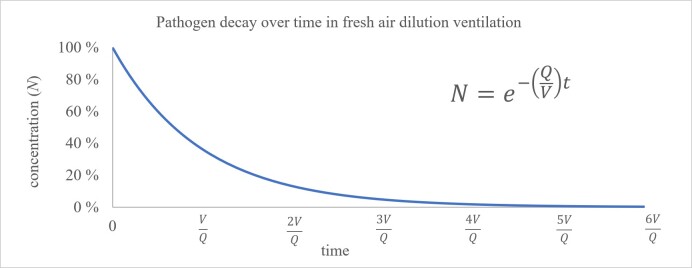
Pathogen concentration decay in response to fresh-air dilution ventilation.

In fresh-air dilution ventilation, a stream of outdoor air that is presumed to be free of pathogens is blown into a space as part of a building's heating ventilation and air-conditioning (HVAC) system and via natural ventilation through doors and windows. The fresh air thus introduced mixes with the air already present in the space, and an equal amount of this mixed air is expelled back outdoors. In practice, it is assumed that the air in a space is completely mixed, and the rate of ventilation is often expressed in air- changes-per-hour (ACH). This metric may be considered as the number of times the air in a space "turns over" per hour. In the case where airborne pathogens are present in the space, they are removed along with this mixed air, and this metric can be applied as the pathogen-removal rate, *Q*, divided by the volume of the space, *V*. The concentration of a pathogen in a space is expected to decrease exponentially over time according to this rate, as shown in Fig. 1. Greater exhaust rates will lower the concentration faster, and the resulting decrease in concentration has diminishing returns as the concentration approaches zero. In dilution ventilation, the pathogen-removal rate is simply the volumetric flow rate of fresh air being introduced into the room. When considering alternative removal methods such as filtration and UV-C inactivation, it is convenient to compare their impact in terms equivalent to fresh-air ventilation. This may be considered as the rate of pathogen-free air delivered to a space even if there is no actual fresh air being added (*e.g.*, when using UV-C disinfection procedures instead of fresh-air ventilation) [5].

## 4 UV-C Inactivation of Airborne Pathogens

The ability of certain wavelengths in the ultraviolet spectrum to disinfect air and surfaces by inactivating microorganisms has been observed for well over a century [6]. It is now understood that this germicidal radiation disrupts the genetic reproduction of these organisms, and that every pathogen has a characteristic susceptibility to inactivation tied to the specifics of its genotype. For a given pathogen, each wavelength in the germicidal band has its own characteristic relative germicidal strength, which is captured in the germicidal effectiveness curve and varies from wavelength to wavelength in a similar fashion across pathogens. While maximum germicidal strength is known to occur at 265 nm, in practice, 254 nm radiation produced by mercury vapor lamps has been the most widely available and applied wavelength, and experimental results are often standardized to this ubiquitous wavelength.

### 4.1 First-Order Decay Equation

UV-C inactivation is modeled according to the first-order decay relationship presented in Eq. (2). It shows that the concentration of a pathogen exposed to a given amount of optical irradiance, *E*, will logarithmically decay over time, much like the concentration of a pathogen in a space will decay because of fresh-air dilution ventilation [7]. In the case of UV-C inactivation, the portion of the exponent in the equation equivalent to ACH (*Q*/*V*) in dilution ventilation is the irradiance expressed in optical power per unit area multiplied by the susceptibility, *Z*, of the organism to the wavelength applied, expressed in unit area per unit energy. This equivalence is the basis of germicidal engineering for air disinfection, but it is only applicable for a single irradiance value. This is limiting in practice, because a range of irradiance values exists simultaneously in a space, and air flow within the room will carry pathogen particles across this varying field over time. Much like the well-mixed room assumption is applied in fresh-air ventilation modeling, a well-mixed condition is often assumed in germicidal engineering, and the average irradiance of the volume is used to predict the resulting bulk inactivation. When measuring the susceptibility of a pathogen, a uniform irradiance field is used to eliminate the impact of this simplification.

N = e^-(EZ)t^ (2)

While the exponential relationship in Eq. (2) is a reflection of natural phenomena, we know that susceptibly in practice occurs in multiple distributions across a population of pathogens, and that it is impacted by environmental conditions [8]. Additionally, pathogens exist in the air in different size particles of variable compositions, which may impact the inactivation mechanisms at play. While the idealized first- order equation is used in this model, the mechanisms at play in practice are complex and difficult to characterize. As such, empirical benchmarks and validation have historically been critical in the application of UV-C disinfection systems and are expected to remain so. However, by exploring the mathematical implications of the first-order equations, we can build insight on the expected relationship between variables and guide empirical investigation and system design.

### 4.2 Capacity Model

**Fig. 2:**
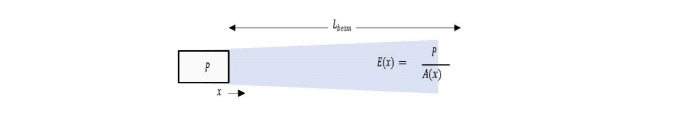
Representation of a propagating wave front without transmission losses where P is the optical power of the source, x is position along the beam length (L_beam_), A(x) is the beam area at position x, and E(x) is the average irradiance of the beam section at position x.

From Eq. (2), the ability of a germicidal system to create irradiance throughout a space will drive the germicidal impact for a given pathogen. Here, we propose to term the sum of irradiance across a volume as "capacity," much like volumetric flow rate is the sum of turnover rate in air-changes-per-unit-time across a volume. This represents the ability, or capacity, of a germicidal system to cause inactivation in a given volume. At the dimensions of concern (1 m to 20 m), UV-C radiation will exit a source and travel through open air effectively unimpeded until it hits an opaque surface because there is very low transmission loss through environmental air at typical conditions [9]. Accordingly, in this model, we consider that power is conserved along a propagating wave front as shown in Fig. 2.

The "optical capacity (OC)" of an irradiance field may be considered as the sum of the irradiance (power per unit area) across a volume and will have units of power multiplied by unit length. It may be evaluated as a bulk property by multiplying average irradiance by volume or by integrating a spatially variable irradiance field across a volume as shown in Eq. (3). It may also be considered as the optical power multiplied by the beam length, also derived in bulk form with an average beam length, or as an integral across the power as shown in Eq. (4). Both forms will constitutively result in the same value. This may be demonstrated by considering the volume integral of the irradiance expression in Fig. 2 as is shown in Eq. (5).

$Optical\;Capacity\;\left(Wm\right)=E\left(\frac{W}{m^{2}}\right)\times V\left(m^{3}\right)=ʃE\;dv$(3)

$Optical\;Capacity\;\left(Wm\right)=P\left(W\right)\times L\left(m\right)=ʃP\;dL=ʃL\;dp$(4)

$ʃE\;dv=ʃ\frac{P}{A\left(x\right)}dv=ʃ\frac{P}{A\left(x\right)}A\left(x\right)dx=PL$(5)

While OC is a useful metric for quantifying germicidal systems, it becomes particularly practical when it is combined with pathogen susceptibility to result in a term "germicidal capacity (GC)," as shown in Eq. (6). GC is in the units of volumetric flow rate and may be directly compared to flow rate in fresh-air dilution ventilation when applied uniformly, as it will have an equivalent impact on pathogen concentration. Because susceptibility is expected to be consistent across a volume, it may be multiplied as a scalar value to the OC integral.

$Germicidal\;Capacity\;\left(\frac{m^{3}}{s}\right)=OpticalCapacity\;\left(Wm\right)\times Susceptibility\left(\frac{m^{2}}{J}\right)$(6)

In practice, there are spatial gradients in the irradiance field produced by divergent radiation sources, which combine with air flows to achieve a distribution of doses across the pathogens in the volume and result in a reduced amount of disinfection relative to the uniform case. The term "disinfection capacity (DC)" is introduced to represent the level of pathogen concentration reduction that is achieved in practice. It is conveniently defined in Eq. (7) as the GC multiplied by a subunity scalar termed "delivery efficiency (DE)."

$Di\text{sin}fection\;Capacity\;\left(\frac{m^{3}}{s}\right)=Germicidal\;Capacity\;\left(\frac{m^{3}}{s}\right)\times Delivery\;Efficiency\left(\%\right)$ (7)

An ideal situation where the volume is uniformly dosed would have a 100% DE, and cases with less mixing and greater gradients would have lower efficiencies. This is because of the diminishing returns associated with the exponential decay relationship. As the concentration of a pathogen approaches zero, an additional unit of optical dose will result in less reduction in pathogen concentration than the same unit of dose applied previously. So, a situation where some sections of a volume receive a dose greater than others is less efficient than if that dose were applied to the volume uniformly. Equation (8) shows the expected reduction of pathogen concentration corresponding to a given DC. This is a powerful metric because it can used to intuit and design a system for a specified volume and clearance rate in the same way that flow rate is used when sizing ventilation systems. Additionally, a germicidal radiation source, which will have a known characteristic distribution pattern, may be evaluated at a discrete number of typical volumes using a capacity volume integral to find a series of capacity values corresponding to room size. This way, systems may be specified, selected, and sized to an application based on simple metrics

$N=e-\left(\frac{DC}{V}\right)t=e-\left(\frac{GCxDE}{V}\right)t$ (8)

**Fig. 3:**
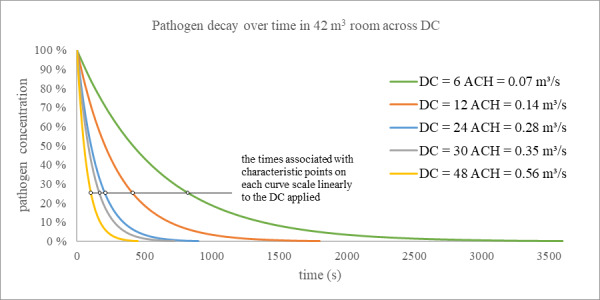
Decay expected in a 42 m^3^ volume associated with various disinfection capacities; see Eq. (8).

Figure 3 shows the decay over time expected when various DC values are applied to a given volume.

Each will be logarithmic in nature and trend to a concentration of zero, with greater DCs reducing the concentration faster. The logarithmic curves scale linearly with DC.

## 5 Modeling Delivery Efficiency

The influence of mixing currents and irradiance gradients has long been a focus of research into the efficacy of UV-C inactivation in air [7]. It is understood that air mixing is required to carry pathogens distributed throughout a volume through the portion of the volume that is irradiated, such as with upper- room ultraviolet germicidal irradiation (UR-UVGI), where only the upper portion of the room is irradiated (which is beneficial to avoid exposing any occupants of the room to the radiation). Computation fluid dynamics (CFD) analyses have been successfully used to predict real-world experimental results [10]. In these analyses, the measured spatial distribution of irradiance within a volume is combined with a computer simulation of air flow expected within that space to find the distribution of doses over time, and the corresponding bulk inactivation expected according to the first-order decay relationship. When compared against experimental results where inactivation was explicitly measured, this approach has been shown to be an effective predictor [10], which further demonstrates the value of the first-order decay relationship as a model for inactivation.

While is it possible to know the spatial variation of irradiance in a deployment through characterization and simple modeling, applying CFD to predict air flow is a complex process requiring accurate inputs and modeling of many contextual factors. This complexity prohibits it from becoming part of the deployment workflow of most UV-C disinfection systems.

In the model presented here, the first-order decay model is also used to predict the delivery efficiency of an irradiance field. This approach involves (1) modeling how a known irradiance field will result in total inactivation over time in a static environment, and then (2) defining a characteristic time associated with a given level of mixing, followed by (3) using this time to evaluate the static efficiency expression as a predictor of overall efficiency. While this approach is expected to be fundamentally less accurate than a CFD approach, it has the unique benefit of only requiring knowledge of the spatial irradiance pattern and a single mixing value, which may be correlated to characteristic settings using more complex approaches.

This is aligned with existing methodologies used in building technology workflows such as building codes and standards where complex engineering relationships and empirical knowledge are made available in simple form in published values and best practices. Furthermore, the closed-form nature of the mathematical expressions guides optimization and insight by allowing the relationships between variables to be explicitly analyzed and intuited. This allows manufacturers to develop better radiation sources and installers to implement more efficient deployments.

### 5.1 Inactivation in an Unmixed Room

We start by considering a static volume with a uniformly distributed pathogen concentration. Although this is not realistic, it allows for an exploration of spatial gradients and inactivation. If there is a uniform irradiance pattern, we expect that the bulk inactivation of the volume will proceed according to the first- order decay relationship in Eq. (2), tied to the uniform irradiance value. Incidentally, we expect this result no matter what the mixing pattern is because all the airborne pathogens will get the same dose no matter how they are distributed through the room.

**Fig. 4:**
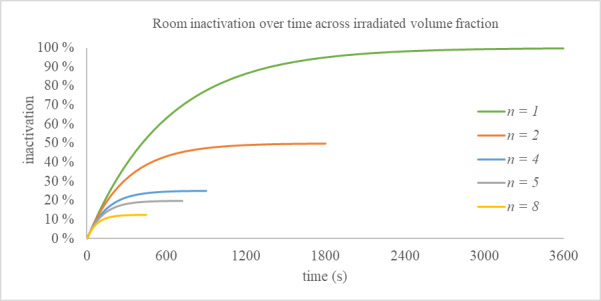
Inactivation expected over time for different irradiated volume fractions, where *n* corresponds to the denominator of the fraction; see Eq. (9).

We can then divide the volume into two discrete sections of known size and consider the case in which only one of the sections has a uniform irradiance applied, while the other receives no irradiance at all. The pathogen concentration in the irradiated zone is expected to decay over time, approaching zero, while the pathogen concentration in the unirradiated section will remain at its starting level. The bulk concentration reduction in this case is limited to the volume fraction of the irradiated zone compared to the total volume as shown in Eq. (9). Figure 4 shows the inactivation expected over time for different irradiated volume fractions where the applied "capacity" is consistent, and *n* corresponds to the denominator of the volume fraction.

$inactivation=\frac{1}{n}\left(1-e\frac{nGCt}{v}\right)$(9)

Taken on its own, the behavior shown is intuitive but irrelevant because of mixing currents. However, the approach-considering the inactivation associated with sections of the volume with uniform irradiance independently and then adding them together to find bulk inactivation-is important. Equation (10) shows how a discrete number of sections may be considered to predict total inactivation, and Eq. (11) shows the limit of this approach as a differential expression where the section being considering is a differential volume at a point in space. This form can explicitly predict the bulk inactivation over time of a pathogen subject to a known spatial irradiance pattern. Furthermore, the inactivation integral for a spatial distribution may be evaluated and equated to a characteristic volume fraction being uniformly irradiated as shown in Eq. (12). This is helpful because it allows for a complex distribution to be expressed as a single value and the implications to be intuited.

$inactivation=\frac{\left(1-e^{{\mathrm{-E}}_{1}Zt}\right)\frac{V}{n}+\left(1-e^{{\mathrm{-E}}_{2}Zt}\right)\frac{V}{n}+....\left(1-e^{{\mathrm{-E}}_{n}Zt}\right)\frac{V}{n}}{V}$(10)

$inactivation=\frac{\int \left(1-e^{\mathrm{-E}Zt}\right)dv}{V}$(11)

$\frac{1}{n}\left(1-{\left(e^{-\frac{GCt}{V}}\right)}^{n}\right)=\frac{\int \left(1-e^{\mathrm{-E}Zt}\right)dv}{V}$(12)

### 5.2 Converting Inactivation to Equivalent Ventilation (Capacity)

Prediction of pathogen inactivation over time, as is done in Eq. (10), Eq. (11), and Eq. (12), has limited usefulness in system design efforts. As discussed previously, equating a resultant amount of inactivation to the equivalent amount of fresh-air ventilation (capacity) allows for a direct comparison of different interventions and for evaluation of expressions such as the Wells-Riley equation. Equation (13) shows how the resulting inactivation predicted by Eq. (11) may be equated to that expected from fresh-air ventilation, and Eq. (14) shows this expression solved for equivalent flow rate. This equivalent fresh-air rate is the DC of the system and may be divided by the GC to find the corresponding DE, as shown in Eq. (15).

$inactivation=\frac{\int \left(1-e^{EZt}\right)dv}{V}=1-e^{-\frac{Qt}{V}}$ (13)

$Q_{equivalent}=DC=-1\times \frac{V}{t}\times \text{In}\left(1-\frac{\int \left(1-e^{EZt}\right)dv}{V}\right)$(14)

$DE=\frac{DC}{GC}=\frac{-1\times \frac{V}{t}\times \text{In}\left(1-\frac{\int \left(1-e^{\mathrm{-E}Zt}\right)dv}{V}\right)}{Z\int Edv}$(15)

### 5.3 Spatial Efficiency Curve

**Fig. 5:**
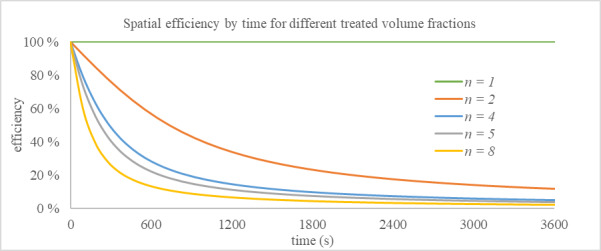
Spatial efficiency curves for different irradiated volume fractions with a uniform GC.

Equation (15) is notably a time-variant expression. This is intuitive when considered in context of the static condition being evaluated. At time zero, there is uniform pathogen concentration in the space, and all portions of the volume have received a uniform dose of zero. However, as time progresses, the variability of dose builds, and the impact of nonuniform inactivation has a greater effect on the efficiency of inactivation. We term the relationship between DE and time as the "spatial efficiency curve." It is a representation of ways in which the spatial nature and power of the irradiance field will drive efficiency at different characteristic times. Figure 5 shows the spatial efficiency curves for different irradiated volume fractions with a uniform GC. 

### 5.4 Establishing a Mixing Coefficient

**Fig. 6:**
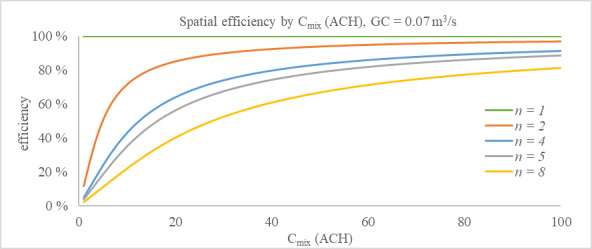
Spatial efficiency curves plotted against the mixing coefficient (*C*_mix_); see Eq. (15).

Knowing the spatial efficiency over time for a static condition has limited usefulness in real-world applications because there are always mixing currents present. However, the expression provides important insight into the ways in which different mixing conditions may couple with spatial efficiency to result in a total inactivation. It is intuitive that situations with very high levels of mixing will carry particles through the field quickly, and there will be less opportunities for the spatial variation of the irradiance field to drive uneven dosing, and *vice versa*. The characteristic time associated with a well-mixed situation will be less than that of a lesser mixed one. It is convenient to define a "mixing coefficient (*C*_mix_)," in the units of 1 over unit time, which is the inverse of the characteristic time used in the spatial efficiency expression. The spatial efficiency curve may then be plotted relative to *C*_mix_ instead of time, as is shown in Fig. 6.

Compared to the approach used in CFD methods, where a single particle is tracked as it moves through an irradiance field, this approach tracks the turnover of particles in a single location within said field. If a volume is mixed with a characteristic mixing velocity, *C*_mix_ could be considered as some function of the mixing velocity and linear dimension of the room because this is a representation of the rate at which the air is cycling through the space. A three-dimensional space would importantly have three different mixing velocities and characteristic lengths corresponding to the three dimensions, but it is possible to combine those into a single value for simplicity's sake or to focus on the dimensions where the spatial gradients are greatest. While this definition of *C*_mix_ is not based on a specific constitutive relationship, it is reasonable to expect that there is a characteristic relationship between the dimensionless parameters that may be representative of behavior across a variety of conditions. This is similar to how the Buckingham π theorem has been used to develop models of physical phenomena across scientific disciplines [11]. Most importantly, it allows for the potential to tie different space and setting characteristics to *C*_mix_ in a way that can be codified and referenced in an application standard. If we assume a first-order relationship between *C*_mix_ and the ratio of mixing velocity and room dimension, we get Eq. (16), where *X* is the scalar coefficient.

$C_{mix}\left(\frac{1}{s}\right)=X\frac{V_{mix}\left(\frac{m}{s}\right)}{L_{room}\left(m\right)}$(16)

### 5.5 Scaling of Spatial Efficiency Curves

**Fig. 7:**
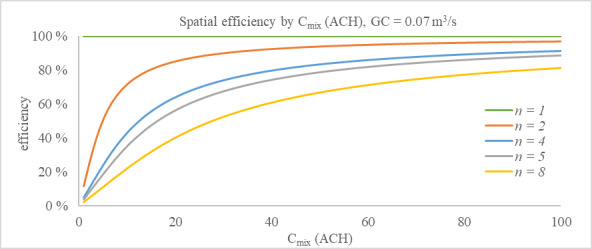
As GC is scaled across consistent spatial patterns and equivalent volume fractions, the spatial efficiency curves will scale proportionally in time.

**Fig. 8:**
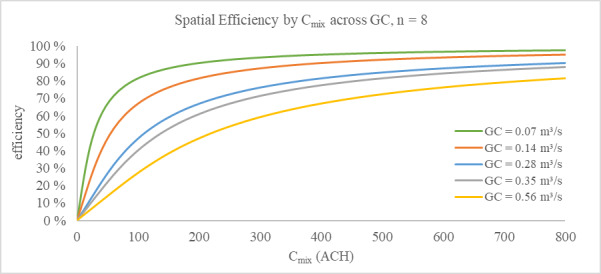
Spatial efficiency curve for different GCs for a given spatial pattern and equivalent volume fraction.

One of the benefits of maintaining closed-form expressions for DE and equating the spatial efficiency integral to a single volume fraction is that the spatial efficiency curve may be used to explore the effects of adjusting the parameters of the characteristic irradiance field. We expect all curves to follow a logarithmic decay, so the resulting scaling of the curves may be used to draw conclusions relative to the interdependencies of the variables. Figure 7 shows that as GC is scaled across consistent spatial patterns and equivalent volume fractions, the spatial efficiency curves will scale proportionally. This means that greater germicidal capacity applied to a volume will require proportionally greater amount of mixing to maintain efficiency, or *vice versa*. Figure 8 shows how the spatial efficiency curve is expected to scale across different GCs for a given spatial pattern and equivalent volume fraction. This relationship would be expected if the characteristic power of a source is modulated, or a pathogen with a different susceptibility is considered.

### 5.6 Use with Enclosed UV-C Air Cleaners and In-Duct Disinfection

Thus far, the relationships explored have been applied generally to an arbitrary volume and irradiance/flow fields. In practice, UV-C disinfection is applied across several modalities, including UR- UVGI, where the unoccupied section of the room above occupants' heads is irradiated, continuous disinfection approaches, where the occupied sections of the room are irradiated, as well as enclosed UV solutions, where a stream of air is passed through an enclosed irradiated volume. The relationships defined here can be applied to all these cases. The case of enclosed UV-C systems is particularly of interest because the nature of the solutions is such that they may be modeled in closed form separately from the expressions derived here with a high degree of confidence. In particular, the volume of an enclosed UV-C solution is generally much less than the total room size, and the fluid dynamics of the air stream within the irradiance field is turbulent in nature to the extent that it is reasonable to treat air flow uniformly. Under these assumptions, the expected impact of the intervention may be deterministically modeled and then compared to the expressions being presented here.

**Fig. 9:**
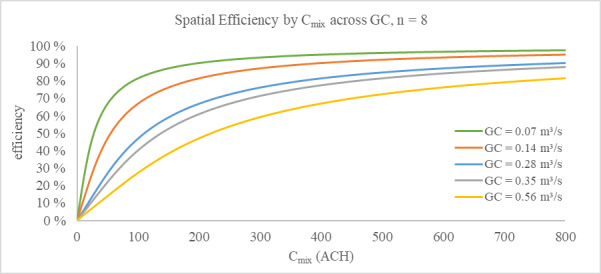
Schematics of an enclosed UV-C air cleaner.

Figure 9 shows a schematic diagram of an enclosed UV-C solution where air with a flow rate, *Q*, is passed through an enclosed volume, *V*_C_, subjected to a uniform irradiance, *E*. From the definition of GC, we may express it as irradiance multiplied by volume or optical power multiplied by average beam length. Also, we know that the dose applied to the air stream will be the residence time in the chamber multiplied by the irradiance. Equation (17) shows how this relationship may be used to predict the resulting inactivation of pathogens in the air stream. Multiplying this inactivation as a percentage times the flow rate represents the amount of fresh air delivered to the space, which is the DC, and if divided by the GC, the resulting value is the DE, as shown in Eq. (18). This is a convenient expression with which to consider the effect of an enclosed UV-C solution. It shows that the efficiency of an intervention is driven by the ratio of the volumetric flow rate to the GC.

$Q_{equivalent}=Q_{flow}\left(1-e^{-\frac{EVZ}{Q_{flow}}}\right)=Q_{flow}\left(1-e^{-\frac{QC}{Q_{flow}}}\right)$

(17)

$Delivery\text{A }Efficiency=\frac{Q_{flow}}{GC}\left(1-e^{-\frac{QC}{Q_{flow}}}\right)$ (18)

We can then take the general-purpose form of DE as shown in Eq. (15) and evaluate it for the case of an enclosed UV setting where the characteristic time is the enclosed volume divided by flow rate, resulting in Eq. (19). While this expression in its closed form is different from Eq. (18), solving numerically shows that the expressions are equal for the range of interest. This correlation is an indication of the validity of the general-purpose delivery efficiency expression from a mathematical perspective.

$Delivery\text{A }Efficiency=\frac{-1\times Q\times \frac{v_{R}}{v_{C}}\times \text{In(1-}\left(1-e^{-\frac{GC}{Q}}\right)\frac{v_{C}}{v_{R}})}{GC}$

(19)

In-duct disinfection systems are expected to behave similarly to air cleaners, with the difference that the air stream in a ducted system is potentially supplied to and exhausted from multiple spaces concurrently and mixed with some fraction of fresh air. Nevertheless, the efficiency expression should still hold when applied to the recirculated fraction, and the delivery efficiency of the GC is related to its proportion of volumetric flow rate. A filter may be present in both HVAC systems and air cleaners, which can also be quantified as a capacity. In that case, the sum of the germicidal capacity and filter capacity should be used in the expression.

### 5.7 Correlation with Existing Experimental Data

Any model for germicidal efficacy must be compared against real-world experimental results to validate its relevance. However, measuring quantitative germicidal efficacy is a difficult task because of the methods needed to quantify pathogen concentration with and without a UV-C intervention. Current best practices require culturing pathogens to evaluate the percentage that remain viable, which is an onerous measurement to carry out with many potential noise sources. However, there are three publicly available data sets in the published literature from studies in which this process was carried out across different parameters [7, 12, 13]. Two were carried out in a test chamber at the Harvard School of Public Health, and one was done in a chamber at the University of Colorado (UC) Boulder. Each one of these live-organism experiments was coupled with a corresponding effort to characterize pathogen susceptibility in uniform irradiance conditions [14-17]. Each effort varied the characteristics of the mixing and irradiance applied while tracking the resulting total inactivation. We took the documented characteristics of the trials and used them to evaluate the GC and general-purpose DE expressions and compare the predicted results to measured results. Numerical data from the comparisons discussed below are tabulated in Appendix A (Sec. 8).

**Fig. 10:**
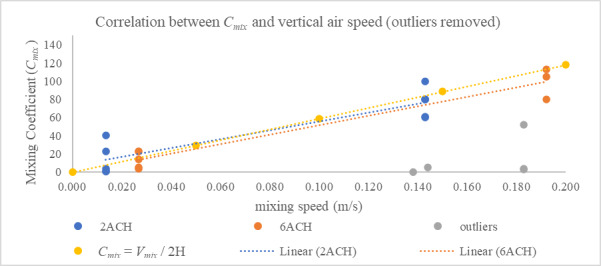
Measured *vs*. calculated mixing coefficient for data from First and Rudnick [7].

In First and Rudnick [7], both irradiance and vertical air speed were modulated and recorded, which make the results particularly useful for testing the model presented here. Figure 10 shows the calculated mixing coefficient required for the DE expression to match the measured results plotted against the measured mixing speed. Also shown is mixing coefficient calculated according to Eq. (16), where the scalar value, *X*, is 1/2. We see a good correlation between the experimental trend lines and the calculated mixing coefficients, which at the very least justifies further exploration of the relationships. Note that only the average irradiance value in a known upper-room volume fraction is available to calculate the spatial efficiency. It is expected that if the gradients of the irradiance field were accounted for, the spatial efficiency would be less, and the calculated mixing coefficients would be greater. A scalar value of *X* up to two would be intuitive, considering there is an equal amount of air moving up and down at once. To confidently test the relationships proposed here, the complete irradiance field should be considered, not just the average upper-room irradiance.

**Fig. 11:**
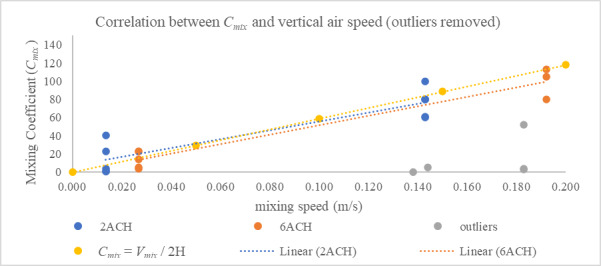
Calculated mixing coefficients from Miller (2002) [12].

In Miller [12], irradiance and pathogen were the primary variables explored. Except for two configurations where no mixing conditions were induced, all experiments involved a baseline level of mixing. In three cases, there were greater than 100% efficiencies measured, which requires further investigation. Figure 11 shows the calculated mixing coefficients across organisms and mixing conditions. In the no-fan configurations, a low mixing coefficient was calculated as expected. In the other cases, there were low mixing rates calculated even when mixing did not follow the model well. Perhaps, in these cases, there was another factor inhibiting inactivation or affecting measurement. For *Mycobacterium bovis BCG* (BCG), very high mixing coefficients were measured.

**Fig. 12:**
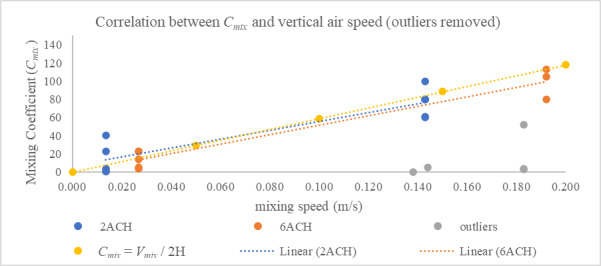
Calculated mixing coefficients from McDevitt *et al.* [13].

**Fig. 13:**
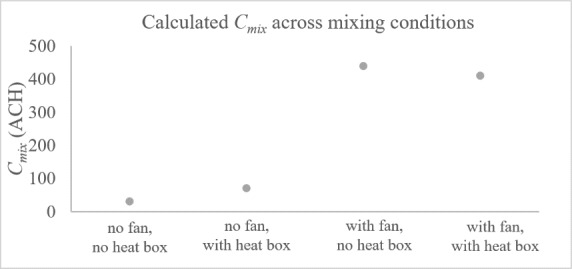
Calculated mixing coefficients across mixing conditions from McDevitt *et al*. [13].

In the study by McDevitt *et al.* [13], all tests were carried out on Vaccinia virus that has a relatively high susceptibility of >2.5 m^2^/J, depending on relative humidity (RH), which is 10 to 100 times that of the organisms used in the other experiments. This results in very high comparative GCs. In the model presented here, this high susceptibility results in spatial efficiency curves that require a high *C*_mix_ to achieve even modest DEs. Figure 12 shows the mixing coefficients corresponding to the measured inactivation across summer and winter conditions. These values are significantly greater than those observed by First and Rudnick [7], even though they were carried out in the same chamber with presumably similar mixing velocities. On its face, this fact would suggest that there is some dependency on GC in the mixing coefficient relationship. When mixing conditions were directly modulated, the mixing coefficients varied as expected, as shown in Fig. 13. Susceptibility values correlating to 50% RH in summer and 40% RH in winter were used.

### 5.8 Testing and Validating the Model

Current CFD approaches that model inactivation of combined flow and irradiance fields have been validated against real-world results, which means they may be used as a first point of reference when evaluating the results of the model presented here. The benefit of a computer simulation is the ability to run many different configurations with a single model to test behavior across a range of variables, as opposed to a physical experiment, where the number of test configurations is more constrained

**Fig. 14:**
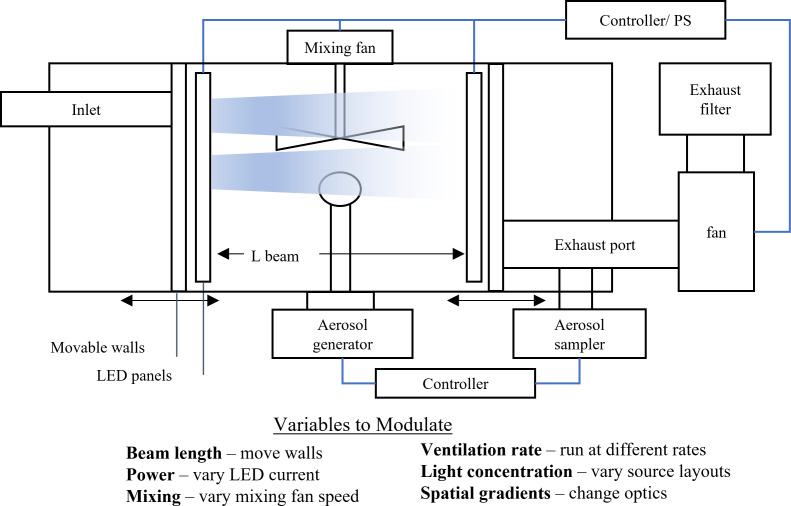
Proposed experimental chamber and variables for exploring validity of expressions derived herein.

However, empirical validation is also a critical step in the validation of the model presented. A series of experiments may be conceived to explicitly evaluate the expressions and relationships empirically by comparing the calculated results against measured results. Notably, this may be carried out in a small-scale chamber for the sake of convenience to explore many variations, and then at real-life scale for a smaller number of conditions to explore the impact of scale. Figure 14 shows a schematic diagram of a chamber that may be used to carry out these experiments and candidate variables to modulate.

## 6 Implications and Use

Currently, there is not an established metric by which to design and compare UV-C disinfection systems for their efficacy, which contributes to market confusion. The capacity model here allows for germicidal engineering calculations to be carried out simply by a variety of stakeholders and persons using precalculated integrals and look-up tables. Just like fan flow rate is used to size HVAC and filtration solutions, DC may be used to find the correct size of a germicidal disinfection system for a given application with a small number of characteristic parameters. By contrast, current best practices in upper- room UVGI engineering recommend an average upper-room irradiance value or a volume-based optical power scaling. None of these approaches captures the impact of spatial gradients in the irradiance field or allows for a simple modulation of pathogen or the resultant pathogen clearance rate, as is possible with a capacity model. While the empirical demonstration of germicidal efficacy in test chambers and real-world applications has been a critical step in demonstrating the effectiveness of UR-UVGI, the use cases for which these benchmarks have been established are for critical care settings with relatively high target clearance rates, where cost pressures are different from broader commercial applications. In addition, they are generally targeted toward bacterial pathogens such as *Mycobacterium tuberculosis*, which have lower susceptibility values than the most ubiquitous viral threats, SARS-CoV-2 and influenza strains. From the spatial efficiency model, it may be appreciated that the OC density in an application relative to the pathogen susceptibility has a strong impact on the DE achieved. This understanding will allow for the sizing and deployment of germicidal systems capable of achieving impactful clearance rates with a high degree of DE at a minimized cost. Moreover, the spatial efficiency integral demonstrates how continuous disinfection approaches like far-UV, where a greater portion of the room is irradiated, may have a relative advantage, optical-watt to optical-watt, when compared to UR-UVGI systems, which would offset the relatively higher cost per watt. Similarly, it demonstrates how direct irradiation below exposure limit (DIBEL) technology [18] can achieve very high clearance rates at relatively low irradiance values.

## 7 Conclusion

The costs of airborne infectious disease transmission are far-reaching, and there is a clear and present need for improved air safety in shared indoor spaces. High-risk settings and particularly risk-sensitive entities have utilized high mechanical ventilation rates, high-efficiency particulate absorbing (HEPA) filtration, and UV-C disinfection for decades to combat transmission risk. There is an opportunity and need to implement these solutions at a large scale wherever people gather to make our societies more resilient to the pathogenic threats that are bound to occur. It is reasonable to imagine a future where a strategic capacity for efficient UV-C disinfection, ready to be utilized when risk is elevated, is a basic feature of the built environment, with additional capacity available for temporary deployment when risk is extremely high. For this future to occur, decision makers must be incentivized to make the investment in equipment, and the benefit and costs need to be quantified and justified. For reliable deployment and maintenance, an easy-to-use framework and language should be available and understandable for those designing, selling, installing, commissioning, maintaining, and buying the equipment. There also needs to be a strategic effort to capture the knowledge and insight of public health experts and scientists relative to risk dynamics and create robust bridges between that insight and its real-world application.

The models and concepts presented here can help build the tools that will enable such a future, but first they must be refined and validated. A pathogen distribution integral expression can potentially be integrated to account for a variable pathogen concentration in space, and the mixing coefficient relationship needs to be explored further. Also, standardized methods to capture spatial data and evaluate integrals should be developed.

## Figures and Tables

**Figure:**
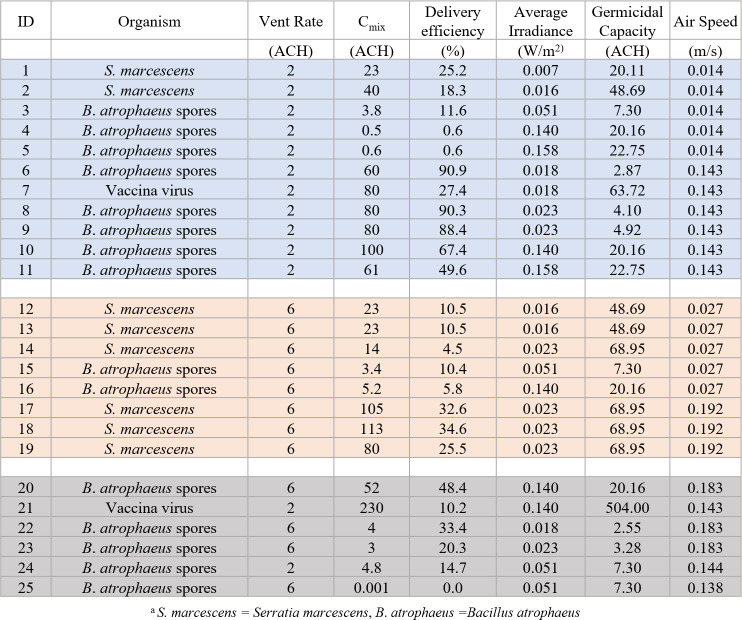
**Table 1.** Tabulated data from First and Rudnick [7].

**Figure:**
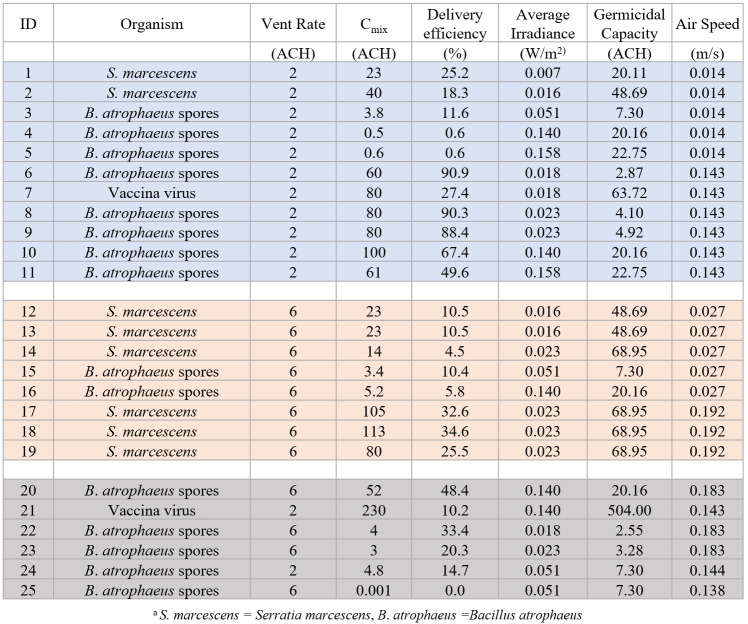
**Table 2.** Tabulated data from Miller [12].

**Figure:**
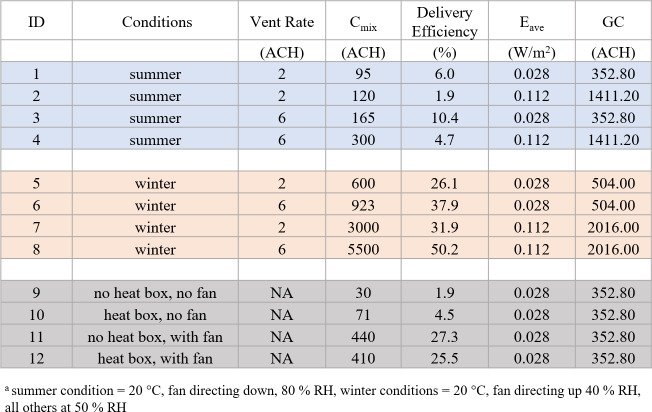
**Table 3.** Tabulated data from McDevitt *et al.* [13].

**Fig. 15:**
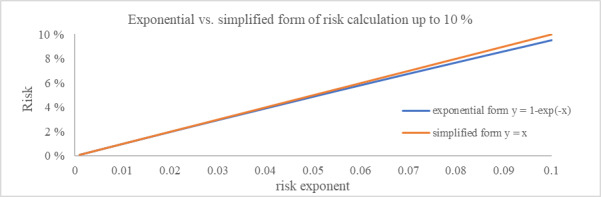
Comparison of exponential and simplified forms of risk expression showing good correlation up to 10%.

**Fig. 16:**
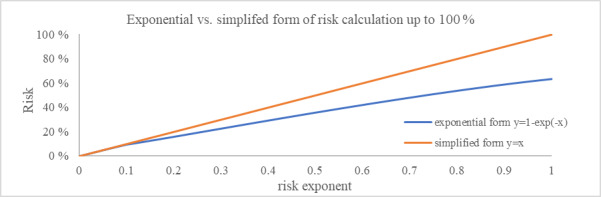
Comparison of exponential and simplified forms of risk expression showing significant divergence at higher risk values.

**Fig. 17:**
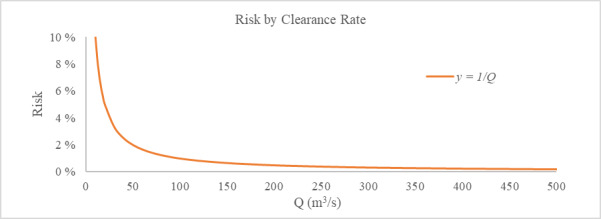
Variation of risk expression across *Q* values for a numerator value of 1; see Eq. (27).

**Fig. 18:**
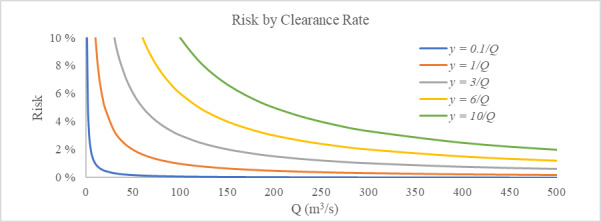
Variation of risk expression across *Q* values for a range of numerator values; see Eq. (8).

## 8 Appendix A-Tabulated Experimental Data

Tabulated data from First and Rudnick [7] is shown in Table 1, data from Miller [12] are shown in Table 2, and data from McDevitt *et al.* [13] are shown in Table 3.

## 9.1 Overview

A form of the Wells-Riley equation is presented here that allows for the comparison of costs associated with disease transmission against the cost of solutions used to prevent such transmissions. Using first principles, an optimal pathogen clearance rate is calculated as a function of relevant variables associated with transmission risk. This approach provides insight for the sizing and operation of equipment and is applicable across air-cleaning modalities, including fresh-air dilution ventilation, filtration, and UV-C inactivation. The integration of such an approach into a broadly applicable control system is discussed.

## 9.2 Introduction

Ventilation in indoor spaces is needed to exchange CO_2_, remove pollution, and prevent aerosol transmission of communicable diseases. Optimally sizing a ventilation system to prevent transmission risk is challenging because of the variability of threat level and the distributed nature of the costs of transmission. It is clear that when an infectious person is present in a shared space, removal or inactivation of exhaled pathogens through ventilation may significantly reduce the likelihood that others in the space will become infected. However, many business owners do not feel like they have enough information about the threat faced and options available to confidently invest in ventilation. There is an opportunity to use scientific understanding of transmission dynamics and build a control framework that identifies the optimal operating point, maximizes return on investment for a business, and automates system function. Such a model relies on accurate information about disease dynamics and incidence, which is available from research and public health institutions. Here, we explore first-principles-based risk modeling expressions and utilize them to compare the cost of ventilation against the cost of transmission in a form that enables an optimal operating point to be identified for a system. We then use this insight to conceive of a protocol for managing the deployment and automation of such a system and discuss the required inputs needed for effective operation.

## 9.3 Wells-Riley Equation

The Wells-Riley equation is the foundational relationship used in airborne transmission risk modeling. Introduced in 1970s, it predicts the likelihood that a susceptible individual will become infected in a shared space based on the number of infectious individuals present (*I*), the rate of inhalation (*p*), the rate of pathogen clearance (*Q*), and a term called quanta generation rate (*q*), as shown in Eq. (20).

$P=\frac{T}{n}=1-e^{\frac{-Iqpt}{Q}}$(20)

Quanta was introduced by Wells to represent a quantity of pathogen capable of resulting in an infection. It is not measured explicitly but rather calculated based on the outcome of a situation where transmissions and other factors are known. In this way, observed epidemiological trends may be used to develop metrics that predict the outcome of other situations [3]. The base form of the expression may be modified based on the specific application to account for different factors such as the use of masks and vaccination rates [19].

If we want to predict risk in a generalized setting, the infection status of room occupants is unknown, so we express the number of infectious individuals in a space as the number of people present multiplied by the infection rate of the general population, as shown in Eq. (21). The population infection rate may be referenced to as specific of a population as is achievable. For instance, it is possible to track infection rate across specific geographical locations as well as within demographics.

$I=n\times i_{pop}$ (21)

In many applications of the Wells-Riley equation, the number of susceptible individuals is often considered to be the number of occupants minus the number of infectors. Because we expect the number of infectors to be much less than 1, given typical community infection rates, and for the sake of simplification of the expression, we do not incorporate this approach and instead consider the number of susceptible individuals to be the total number of room occupants.

Quanta generation rate may be inferred for a specific disease and activity (*e.g.*, talking, singing, *etc*.) by analyzing epidemiological data sets [20]. Additionally, the variable infectiousness of a given dose to an individual or population based on immunity and health factors may be modeled. This is done by including a susceptibility term in the units of infections per quantum [19]. Immunity from vaccination or previous infection may result in a lower susceptibility value, whereas comorbidities and other factors may result in an increased susceptibility. All these factors may be averaged over a population. The impact of mask usage by infectors may also be accounted for by inclusion of a mask factor, as expressed in Eq. (22). This represents the ability of masks to prevent some portion of the quanta generated by the individual from being introduced into the room, and the quanta generation rate is then considered to be that for the unmasked case.

*q* = *q*_no-mask_ × (1 - *η*_mask_) × *S*_pop_ (22)

The pulmonary inhalation rate, *p*, used in Eq. (1) represents the amount of (potentially infectious) air that a susceptible individual inhales. We expect the use of a mask by susceptible individuals to reduce the number of pathogens that are inhaled, so the expression is modified to account for the impact of wearing a mask as shown in Eq. (23).

*p* = *p*_no-mask_ × (1 - *η*_mask_) (23)

Combining Eqs. (20-23) above, we get Eq. (24) as a predictor of risk. We may call the exponent in the expression the "risk exponent" and may further define the "risk coefficient (C_risk_)" as all the variables except for time, *t*, clearance rate, *Q*, and number of people, *n*, as shown in Eq. (25), which combines with Eq. (24) to get Eq. (26).

$P=\frac{T}{n}=1-e^{\frac{i_{pop}\times S_{pop}\times q_{no-mask}\times P_{no-mask}\times {\left(1-\eta_{mask}\right)}^{2}\times n\times t}{Q}}$(24)

*C*_risk_ = *i*_pop_ × *S*_pop_ × *q*_no-mask_ × *P*_no-mask_ × ${\left(1-\eta_{mask}\right)}^{2}$ (25)

$P=\frac{T}{n}=1-e^{\frac{c_{risk}\times n\times t}{Q}}$ (26)

## 9.4 Simplification of Risk Expression

For small values, it is possible to simplify the exponential form of Eq. (26). Figure 15 shows that for values of *P* < 10%, there is a strong correlation between the exponential form (*y* = 1 - exp[-*x*]) and the simplified form (*y* = *x*), while Fig. 16 shows the two forms diverging more greatly at increased risk values. At *P* = 10%, there is only a 5% difference between the two, with better correlation at smaller values.

Because the risk of transmission is likely to be much less than 10% in most settings, we may use this simplification to express Eq. (26) as Eq. (27), which is much more convenient.

$P=\frac{T}{n}=\frac{c_{risk}\times n\times t}{Q}$(27)

## 9.5 Variation of Risk Relative to *Q*

We may now use Eq. (27) to explore the effect of different clearance rates on resulting risk. Figure 17 shows a plot of risk relative to clearance rate, where the numerator in Eq. (27), (*C*_risk_ × *n* × *t*), is equal to one. It shows that the slope of the curve changes significantly across clearance rate. At small values, a small increase in clearance rate results in a large decrease in risk, but at higher values, a large increase in clearance only results in a small decrease in risk. This demonstrates that each operating clearance rate is tied to an incremental risk reduction achievable with added clearance.

Figure 18 shows the risk curves corresponding to different numerator values in Eq. (27), which may be considered as the net risk profile of a setting due to the number of people, length of exposure, and parameters captured in *C*_risk_. The characteristic slopes of the curves are different at a single clearance rate, meaning that the incremental value in terms of risk reduction depends on both the clearance rate and the numerator, (*C*_risk_ × *n* × *t*).

## 9.6 Cost Modeling

Figure 18 showed that there are diminishing returns for added clearance rate in each situation. An optimized risk mitigation strategy will attempt to maximize the reduction in risk achieved for an expenditure of resources, and to do that, we must relate the cost of risk and resources in equivalent units. It is convenient to use monetary value for this comparison. Equation (28) shows that the total cost of transmission over some time, *t*, may be expressed as the number of transmissions, *T*, multiplied by a characteristic cost (*c*_trans_) in the units of dollars per transmission. This could be the value of lost productivity to an employer from a sick employee, and it could also reflect the cost of healthcare associated with treating the disease, the impact to quality of life for the sick individual, or the costs of future transmission events from the newly infected occupant. Clearly, different stakeholders will have different sensitivities and internal cost profiles that may be uniquely captured with this variable.

$C_{trans}=\frac{T\times c_{trans}}{t}$(28)

The cost of added clearance rate may also be modeled. A variety of interventions may be used to clear pathogens from a space, including fresh-air dilution ventilation, filtration, and germicidal inactivation [21]. The effect of each of these may be quantified in consistent units of clean-air volume per unit time, and the operating cost rate of each may be evaluated on a per-unit-flow-rate basis. Each intervention has a fixed cost rate, *C*_fixed_, representing the purchase price of equipment plus the cost of installation, commissioning, and scheduled maintenance amortized over the operational life of the system. *C*_fixed_ is a multiple of the specific per-unit-flow-rate fixed cost rate, *c*_fixed_, and the installed capacity, *Q*_cap_, as shown in Eq. (29). Notably, these costs are independent of the relative utilization of the equipment.

*C*_fixed_ = *c*_fixed_ × *Q*_cap_ (29)

Each intervention also has variable costs, *C*_var_, tied to the cost of consumables (*e.g.*, filters, lamps), unplanned maintenance, personnel operational costs, as well as energy usage. These costs are the multiple of the utilized clearance rate, *Q*_used_, and the specific variable cost rate, *c*_var_, as shown in Eq. (30).

*C*_var_ = c_var_ × *Q*_used_ (30)

Equation (31) shows the total cost of an intervention per unit time, which depends on the capacity available as well as the utilization.

*C*_tot_ = *C*_fixed_ + *C*_var_ = *c*_fixed_ × *Q*_cap_+*c*_var_ × *Q*_used_ (31)

## 9.7 Optimization

With the costs of transmission and clearance rate defined, we may now take Eq. (27) and derive the number of expected transmissions as a function of clearance rate as shown in Eq. (32), which may be combined with Eq. (28) to result in the total monetary cost rate of transmissions as shown in Eq. (33).

$T=\frac{c_{risk}\times n^{2}\times t}{Q}$(32)

$C_{trans}=\frac{c_{risk}\times c_{trans}\times n^{2}}{Q_{used}}$ (33)

We may now use our cost expressions to find the operating clearance rate at which the cost savings achieved by reducing transmission with added clearance rate are equal to the costs associated with that added clearance rate. In effect, this represents the break-even point of an intervention. To do this, we equate the absolute value of the first derivatives of Eq. (31) and Eq. (33) relative to clearance rate as shown in Eq. (34), Eq. (35), and Eq. (36). We may then solve for *Q*_used_, resulting in Eq. (37). This represents the break- even operating clearance rate.

$\frac{d}{dQ_{used}}(C_{trans})=\frac{-d}{dQ_{used}}(C_{tot})$(34)

$\frac{d}{dQ_{used}}(\frac{C_{risk}\times c_{trans}\times n^{2}}{Q_{used}})=\frac{-d}{dQ_{used}}(c_{fixed}\times Q_{cap}{\text{+c}}_{\mathrm{var}}\times Q_{used})$(35)

$\frac{-C_{risk}\times c_{trans}\times n^{2}}{Q_{used}}=-c_{\mathrm{var}}$(36)

$Q_{optimum}=n\times \sqrt{\frac{C_{risk}\times c_{trans}}{c_{\mathrm{var}}}}$ (37)

## 9.8 Selecting Installed Capacity

Note that the cost model presented in Eq. (31) considers both the installed and utilized capacity, while the operating point found in Eq. (37) only establishes an optimum for utilized capacity in response to variable costs. There are several approaches that may be used to find the appropriate optimized installed capacity. One approach would be to take a reference "worst-case" set of risk conditions (*C*_risk_, *n*) and set the installed capacity to match the optimum utilized capacity in this case, where the fixed costs are blended with the variable costs. However, depending on how often worst-case conditions are present, this could lead to an inefficient expenditure of resources. Understanding the expected temporal variation of *C*_risk_ and *n* would allow for use of an averaging approach to find the optimal reference conditions on which to establish *Q*capacity.

The nature of epidemiological trends is that infection rates in a community may vary from season to season and year to year for endemic diseases like influenza or may have noncyclical spikes for novel pathogens like SARS-CoV-2 variants. In addition, local outbreaks of previously eradicated disease may occur [22]. It is often less compelling for a business to invest in protection against a risk that may arise in the future. As such, there may be benefit to temporary capacity that can be installed or accessed quickly when increased community infection rates occur in a specific geographic area or location. This temporary capacity may be shared strategically across a large geographic area, thereby spreading the upfront costs for the equipment across several entities.

## 9.9 Implementation in a Control System

Equation (37) is a powerful insight that provides effective guidance for a challenge that is often difficult to navigate (*i.e.*, determining the ventilation rate at which to operate). It allows for a system that dynamically changes and adapts to evolving facts on the ground. If captured as inputs, changes in room occupancy, community infection rate, and other dynamics can be leveraged to figure out the optimal operating point in real-time and deploy a control system in response. Quanta generation rates, *q*, are known to be particularly variable between pathogens and across situations, and this has historically been a challenge in establishing guidelines [23]. However, there are established workflows to capture these values, and public health experts are continuously gathering and analyzing data to that end [24]. Similarly, infection rate tracking and modeling can be carried out with a high degree of location specificity [25, 26, 27]. A control system is proposed where epidemiological factors are actively obtained from public health agencies and researchers in a controlled, contextual, and collaborative manner. The inputs may be provided across a range of parameters, and then the system selects the most appropriate input based on some knowledge of the situations. For instance, quanta generation rates can be provided for a variety of baseline activities and populations, and the system may select the most appropriate value based on an understanding of the specific situation to which it is being applied.

Situational risk parameters like occupancy, mask usage, and activity may be input from multiple sources. The most straightforward method is manual input, both during commissioning and during ongoing operation. Current digital tools and internet of things (IoT) make this feasible with a relatively simple user experience, but any manual process is reliant on continued compliance, and it is desirable to automate as much of the information gathering as possible. Automation could address compliance issues and compile large data sets, which could be leveraged with machine-learning methods to build insight and accuracy.

Existing building management systems (BMS) can track occupancy through a building with a variety of methods. Any one of these approaches could be used to understand room occupancy, either through a BMS or as a stand-alone capability. Also, CO_2_ concentration above background is regularly used to monitor air- quality conditions and often to guide HVAC operation in demand-controlled ventilation applications. Rudnick and Milton (2003) [28] showed that environmental CO_2_ concentration over background may also be used as an indicator of transmission risk, because it is a composite measurement of source strength (occupancy) and clearance (ventilation), although it does not capture the impact of filtration or UV-C inactivation, making it an imperfect stand-alone value. However, if combined with explicit knowledge of occupancy or fresh-air ventilation, CO_2_ concentration may be used to deterministically infer the other value. It is a relatively simple proposition to gain knowledge of fresh-air ventilation rate by characterizing a space and then monitoring the HVAC system power state through a BMS. Coupled with room CO_2_ concentration, this knowledge allows for control of the clearance rate with a high degree of confidence.

Knowing the most appropriate clearance rate at which to operate for a given situation is only part of a complete solution. The clearance capacity must be available, and the means to modulate said capacity is also required. Pathogen clearance capacity may come from a range of equipment. As demonstrated in the main text, filtration and UV-C inactivation both may be quantified and sized in terms of the equivalent clean air provided. It has been shown that both filtration and UV-C inactivation, and particularly UV-C inactivation, offer significantly lower fixed and variable costs than HVAC capacity [4], suggesting that they are more appropriate interventions as compared to upgrading central HVAC beyond current levels. Modulation of the power state of said interventions once they are in place is a relatively simple task. Current IoT options to accomplish this are readily available, and there are many building-control interfaces currently in operation as precedence for implementation and administration of such intelligent systems.

The resulting historical data that will be generated by such a monitoring and control system have value from a reporting standpoint as well as potential for use in epidemiological analyses.

## 9.10 Incentives and Externalities

One of the challenges in achieving market adoption of ventilation solutions that have an overall compelling value for their cost is that the entity responsible for facilities and infrastructure is just one of many stakeholders impacted and may not be exposed to all the costs of a transmission event. In addition, if said entity is not subject to incentives relative to their own utility, it is not rational for them to undertake the deployment of such a system. This macroeconomic situation results in an outcome where every player acts rationally, but the outcome is still suboptimal. Conceptually, this is a situation where regulations or subsidies may be used to shift the value of the greater good onto the decision-making entity. The system presented here provides a unique opportunity to calculate and administer such a shift through the *c*_trans_ metric and tracking of the risk characteristics.

Additionally, medical insurance premiums are another item that may be used to incentivize behavior because it is in an insurance provider's best interest to minimize the number of transmissions because of the cost of care. It is conceivable that a provider would entertain lower rates for a workplace that can tangibly show a reduction in number of transmissions through optimized capacity implementation.

## 9.11 Glossary of Variables and List of Equations

**Table:**

*Symbol*	*Variable*	*Units*
*P*	*Probability of infection in a room*	(*%*)
*T*	*number of transmissions a room*	(*#*)
*n*	*number of occupants a room*	(*#*)
*l*	*number of infectors a room*	(*#*)
*p*	*effective respiration rate*	(*m*^3^*/s*)
*q*	*effective quanta generation rate*	(*#/s*)
*t*	*time*	(*s*)
*Q*	*volumetric flow rate*	(*m*^3^*/s*)
$i_{pop}$	*infection rate in the population*	(*%*)
$q_{no-mask}$	*no mask wanted generation rate*	(*#/s*)
$I_{mask}$	*mask filtration efficiency*	(*%*)
$s_{pop}$	*susceptibility of the population to infection*	(*#/#*)
$P_{no-mask}$	*no mass respiration rate*	(*m*^3^*/s*)
$c_{risk}$	*risk coefficient*	(*m*^3^*/s*^2^)
$c_{trans}$	*transmission cost rate*	($*/s*)
$c_{trans}$	*specific transmission cost*	($*/#*)
$c_{fixed}$	*fixed cost rate*	($*/s*)
$c_{fixed}$	*specific fixed cost rate*	($*/m*^3^)
$Q_{cap}$	*available flow rate capacity*	(*m*^3^*/s*)
$C_{\mathrm{var}}$	*variable cost rate*	($*/s*)
$c_{\mathrm{var}}$	*specific variable cost rate*	($*/m*^3^)
$Q_{used}$	*flow rate used*	(*m*^3^*/s*)
$C_{tot}$	*total cost rate*	($*/s*)
$Q_{opt}$	*optimal operating flow rate*	(*m*^3^*/t*)

**Table:**

no.	Equation
20	$P=\frac{T}{n}=1-e^{\frac{-1qpt}{Q}}$
21	$I=n\times i_{pop}$
22	$q=q_{no-mask}\times \left(1-\eta_{mask}\right)\times S_{pop}$
23	$p=p_{no-mask}\times \left(1-\eta_{mask}\right)$
24	$P=\frac{T}{n}=1-e^{\frac{i_{pop}S_{pop}\times q_{no-mask}\times P_{no-mask}\times {\left(1-\eta_{mask}\right)}^{2}\times n\times t}{Q}}$
25	$C_{risk}=i_{pop}\times S_{pop}\times q_{no-mask}\times P_{no-mask}\times {\left(1-\eta_{mask}\right)}^{2}$
26	$P=\frac{T}{n}=1-e^{\frac{c_{risk}\times n\times t}{Q}}$
27	$P=\frac{T}{n}=\frac{c_{risk}\times n\times t}{Q}$
28	$C_{trans}=\frac{T\times c_{trans}}{t}$
29	$C_{fixed}=c_{fixed}\times Q_{cap}$
30	$C_{\mathrm{var}}=c_{\mathrm{var}}\times Q_{used}$
31	$C_{tot}=C_{fixed}+C_{\mathrm{var}}=c_{fixed}\times Q_{cap}+c_{\mathrm{var}}\times Q_{used}$
32	$T=\frac{C_{risk}\times n^{2}\times t}{Q}$
33	$C_{trans}=\frac{C_{risk}\times c_{trans}\times n^{2}}{Q_{used}}$
34	$\frac{d}{dQ_{used}}\left(C_{trans}\right)=\frac{\mathrm{-d}}{dQ_{used}}\left(C_{tot}\right)$
35	$\frac{d}{dQ_{used}}\left(\frac{C_{risk}\times c_{trans}\times n^{2}}{Q_{used}}\right)=\frac{-d}{dQ_{used}}\left(c_{fixed}\times Q_{cap}\times c_{\mathrm{var}}\times Q_{used}\right)$
36	$\frac{-C_{risk}\times c_{trans}\times n^{2}}{Q_{used^{2}}}=-c_{\mathrm{var}}$
37	$Q_{optimum}=n\times \sqrt{\frac{C_{risk}\times c_{trans}}{c_{\mathrm{var}}}}$
